# Surface Modification of Carbon Fibers by Low-Temperature Plasma with Runaway Electrons for Manufacturing PEEK-Based Laminates

**DOI:** 10.3390/ma15217625

**Published:** 2022-10-30

**Authors:** Pavel V. Kosmachev, Sergey V. Panin, Iliya L. Panov, Svetlana A. Bochkareva

**Affiliations:** 1 Institute of Strength Physics and Materials Science SB RAS, 2/4, Akademicheskii Pr., 634055 Tomsk, Russia; 2 Department of Materials Science, Engineering School of Advanced Manufacturing Technologies, National Research Tomsk Polytechnic University, 30, Lenina Pr., 634050 Tomsk, Russia

**Keywords:** carbon fiber, PEEK, plasma with runaway electrons, laminates, adhesion, stress–strain state, finite element method (FEM)

## Abstract

(1) Background: The paper addresses the effect of carbon fibers (CFs) treatment by low-temperature plasma with runaway electrons on the deformation behavior of the polyetheretherketone (PEEK)-layered composites. (2) Methods: The effect of the interlayer adhesion on the mechanical response of the composites was assessed through the tensile and three-point bending tests. In addition, computer simulations of the three-point bending were carried out with the use of the finite element analysis (FEM) with varying conditions at the “PEEK–CF layers” interface. (3) Results: DRE–plasma treatment during the optimal time of t = 15 min led to formation of a rougher surface and partial desizing of a finishing agent. The shear strength of the layered composites increased by 54%, while the tensile strength and the flexural modulus (at three-point bending) increased by 16% (up to 893 MPa) and by 10% (up to 93 GPa), respectively. (4) Conclusions: The results of the numerical experiments showed that the increase in the stiffness, on the one hand, gave rise to enlarging the flexural modulus; on the other hand, a nonlinear decrease in the strength may occur. For this reason, the intention to maximize the level of the interlayer stiffness can result in lowering the fracture toughness, for example, at manufacturing high-strength composites.

## 1. Introduction

In recent years, polyetheretherketone (PEEK) has been widely used for manufacturing polymer composites because of its excellent performance characteristics (thermal stability, chemical resistance, impact strength, technological efficiency, recyclability, etc.) [1,2]. PEEK-based composites are widely employed in high-performance applications such as aerospace, medicine, and others [3,4,5,6], however carbon fibers (CF)/PEEK composites have become the most widely applied due to their high-strength properties.

The important aspect in design of the fiber-reinforced composites is ensuring the required structure and a high level of adhesion at the fiber–matrix interface. This allows us to transfer applied external stresses from a binder to reinforcing fibers, and, consequently, to ensure the overall mechanical properties of the composite.

The solution of this problem requires a careful study of mechanisms responsible for interphase bonding and providing a high level of the interlayer adhesion (for the design of layered composites–laminates). The surface of CFs is smooth and chemically inert [7,8]. Simultaneously, there are a few reactive chemical-active groups in the PEEK molecular structure that result in suppressed chemical interaction at the interphase and interlayer boundaries when sintering the CF/PEEK laminates.

Currently, some approaches have been developed for increasing the adhesive interaction between polymer and carbon fibers. Among them there are thermal oxidation, chemical modification, physical treatment, plasma treatment, and some others.

### 1.1. Thermal Oxidation

Thermal oxidation of the CFs’ surface results in the formation of the oxygen-containing groups with a high polarity (-COOH, -OH, -C=O, etc.). This increases the fiber surface wetting and promotes the interfacial bonding with the polymer matrix (binder). Depending on the oxidation modes, the structure and morphology of the fiber surface can vary significantly.

Li, N., et al. [9] studied the effect of the preheat treatment on the surface properties of CFs and the interfacial properties of PEEK composites reinforced by the CF (CF/PEEK). Desizing gave rise to the increase of the interfacial shear strength of the composites by 15.3%. The reason was that the sizing layer (designed for a thermosetting binder) did not provide the high adhesion strength due to a poor chemical bonding between a sizing agent and the PEEK. Kiss, P., et al. [10] proposed the heat treatment method for the CF–fabric aimed at desizing an incompatible agent and providing the interphase adhesion with the thermoplastic matrices due to oxidation of the CF surface. Desizing was conducted with infrared irradiation; the treatment temperature was 400 °C. The thermoplastic composite laminates consisting of CF/PEEK were fabricated with the use of a hot-pressing method. For the IR–radiation desized CFs, the degree of wetting and the interphase adhesion was assessed, while the Scanning Electron Microscope (SEM)-micrographs of the fracture surfaces of the samples after mechanical testing were analyzed. Jin, Z., et al. [11] proposed the method for treating the surface of the CFs with ozone. It was shown that ozone treatment at temperature of 120 °C for 6 min increased the content of carbonyl functional groups and the surface roughness; in doing so, the degree of graphitization of the CF surface was changed, while the wettability was improved. As a result, the compressive and bending strengths of the CF–composites were increased by dozens of percent. Li, J. [12] used the ozone modification and the air oxidation to treat the surface of the CFs. The author underlined that the Interface Shear Strength (IFSS) values of the CF/PEEK composites with the ozone-treated CF increased by 60% as compared to the untreated carbon fiber composites. X-ray Photoelectron Spectroscopy (XPS) results showed that the ozone treatment increased the amount of carboxyl groups on the surface of the CF. Therefore, the interfacial adhesion to the PEEK matrix was effectively improved.

### 1.2. Chemical Modification

Common practice is to use chemical modifiers and powder dispersions for the pre-treatment of fibers. As a result, a transition layer is formed between the fibers and the matrix, which primarily increases the chemical component of adhesion.

Su, Y., et al. [13] prepared and used water-soluble sulfonated polyetheretherketone (SPEEK) to improve the interfacial adhesion in the CF/PEEK composites. The results have shown the increase in free surface energy and the surface roughness of the CF treated by the SPEEK. The formation of the gradient interface was induced by the chemical reaction between the CF and the sizing agent, as well as by the improved compatibility of the sized fiber and the matrix. As a result, the interfacial adhesion was increased. Yuan, C., et al. [14] synthesized an easily purified semi-aliphatic polyimide to use it as a CF sizing agent. The composites with fibers treated by the sizing agent have shown the increase in ILSS by 20%, while the flexural strength improved by 8%. The polar component of the sizing coating increased the surface energy by 28%. Lyu, H. et al. [15] modified the interface between the CF and the PEEK by coating hydroxylated PEEK grafted onto multi-walled carbon nanotubes (M-HPEEK). The surface tended to become rougher, the number of polar groups increased, the surface energy increased as well, and the wettability improved. This enabled the improvement of physical adhesion and infiltration of the PEEK. As a result, the InterLayer Shear Strength (ILSS), the flexural strength, and the flexural modulus of the modified CF/PEEK composites increased by 73%, 163%, and 85%, respectively. Wang, T., et al. [16] analyzed the prospects of using polyimide as a sizing agent for the short CFs, when PEEK–composites were manufactured by the extrusion and injection molding. The 1 wt % polyimide concentration was sufficient to increase the tensile strength and the flexural strength of the corresponding composite by 16.8% and 8.2% as compared to one without a polyimide sizing agent. Yan, T., et al. [17] developed a number of soluble aminated polyetheretherketones (PEEK–NH_2_) for the use as the CF surface modifiers to improve the interphase properties of the CF/PEEK composites. The study has shown the prospects of increasing the interlayer shear strength and the flexural strength of these composites by 43% and 62%, respectively, as compared to the unsized CF composites.

### 1.3. Plasma Treatment

Plasma treatment is one of the most high-tech and environmentally friendly methods of fiber surface modification that has been extensively studied for the last decades [18,19,20]. In particular, a number of thorough reviews on CFs’ plasma treatment have been introduced in [21,22,23,24,25]. Their authors have focused on the key advantages of plasma treatment for ensuring “fiber–matrix” interaction when fabricating advanced engineering fiber-reinforced polymer composites. The treatment implies placing the fiber into an active medium of ionized gas (O_2_, He, N_2_, NH_3_, etc.). As a result of this, the surface is activated: functional polar groups are formed, and the surface energy increases. This leads to improved wetting of fibers by a polymer binder, while the chemical component of adhesion increases [26,27,28]. In addition, plasma treatment leads to purifying contaminants and loosely bound layers. Subsequent ion bombardment creates a more developed surface, which improves a mechanical interlocking with the polymer matrix of the composite [29,30].

Tiwari, S. et al. [31] presented the results of CFs treatment in nitrogen–oxygen plasma. This has improved the interfacial properties in thermoplastic polymer–carbon fiber reinforced composites, including PEEK. The XPS method showed the increase in the intensity of O/C and N/C peaks, which confirms the bonding of oxygen and nitrogen groups on the surface of the CF; these groups are promoters of adhesion to the polymer. Gravis, D. et al. [32] illustrated that the treatment with radiofrequency (RF) plasma caused a change in the physicochemical properties of the CFs. This was confirmed by measurements and calculations of the free surface energy and the corresponding static contact wetting angles. Various plasma-forming gases were investigated. The greatest effect was obtained for O2-plasma, which was due to the increase in the surface energy of the CFs. The optimization of the technological mode of oxygen plasma treatment applied to the CFs allowed Baghery Borooj, M. et al. [33] to increase ILSS of the composites up to 28% as compared to those fabricated from the untreated fibers. However, the effect of increasing ILSS, achieved due to plasma treatment, was simultaneously accompanied by the decrease in the tensile strength. This was caused by the formation of some surface defects [23,34,35].

Ma, K. et al. [36] studied the effect of plasma oxidation on non-equilibrium dynamic adsorption of epoxy resin and hardener onto CFs’ surface. In contrast to the untreated CFs, the epoxy mostly absorbed onto the functional groups on the treated fibers’ surface. This affected the epoxy curing at the interface, as well as the interface “fiber–matrix” adhesion. The increase of the ILSS and the flexural strength was illustrated.

Santos, A.L. et al. [37] conducted a surface modification of the CFs by the atmospheric pressure dielectric barrier discharge (DBD) in order to improve the adhesion between the CFs and the polypropylene (PP) matrix. The 5 min DBD treatment gave rise to the highest ILSS value. However, the 10 min treatment was responsible for the decrease of the shear strength. The authors suggested that the phenomenon was related both to creating the functional groups on the fibers’ surface as well as the roughness decrease and the mechanical strength.

Diblíková, L. et al. [38] studied the geopolymer composite reinforced with the CF. Four types of argon atmosphere were employed for CF surface treatment. The data of some mechanical tests have proven an adhesion increase of argon plasma-treated fibers, while the functional groups were found for all treated fibers. The authors concluded that the nature of the geopolymer matrix hindered soaking of the CF bundles that reduced efficiency of plasma treatment of the unsized CFs.

Z. Károly et al. [39] compared (i) low-pressure RF and (ii) atmospheric pressure DBD plasma treatments for surface modification of the CFs. It was shown that the DBD method was more efficient as compared to the RF–plasma one, even during a short treatment time (10 s). The former increased the numbers of O and N atoms on the CF surface. However, the DBD doubled the roughness due to surface etching. Thus, the tensile strength decreased. The adhesion was expectedly enlarged by 20 и 50% for RF and DBD–plasma treatments, respectively.

Khan, A. et al. [40] invented low-pressure oxygen plasma treatment for CF functionalization named as Atom Transfer Radical Polymerization (ATRP). In this case, the activator was generated by the electron transfer method (AGET). With proper adjustment of the plasma variables, 98% of their original tensile strength of the CFs was retained. The proposed surface-modification technique allowed the improvement of the tensile strength, the tribological properties, and the surface hardness as compared to the use of the pristine CFs.

CF. Xiao, J. et al. [41] substantiated the time dependent mechanisms of CF plasma treatment. At the initial stage (a treatment time less than 15 s), the plasma mostly etches the carbon atoms on the CF surface. In the case, the ILSS improves due to an increase of adhesion. At the later stage (a treatment time above 30 s), the etching of the amorphous carbon slows down. Thus, a chemical modification of the CF’s surface consists of introducing the oxygen-contain groups. The latter provided a continuous increase of the ILSS.

Radjef, R. et al. [42] employed a pilot scale facility for air RF–plasma treatment of the CF surface. The results were compared with a conventional aqueous electrolytic oxidation. It was shown that regardless of the higher oxygen incorporation that resulted from the electrolytic oxidation, the proposed air plasma treatment ensured comparable enlargement of the IFSS. The removal of weakly bonded surface graphite layers, as well as improving surface roughness, were the key factors responsible for promoting the matrix–fiber adhesion. The authors suggested that dry plasma treatment can be alternately employed instead of industrial wet one.

Thus, the results on various fibers’ plasma modification, as well as the characteristics of physical–mechanical properties of the reinforced composites, have been widely described in research papers. To the best of our knowledge, the very limited data are available on DRE–plasma treatment of the CF tapes as a low-cost and time-saving surface modification technology [43,44]. At the same time, most of the papers confess that the intensive plasma treatment results in both creating a developed surface as well as reducing fibers’ tensile strength due to surface layer damaging [18,33]. For this particular reason, the conducted numerical analysis of PEEK/CF laminates is of actuality for establishing quantitative correlation between the interlayer shear strength and the shear strength.

The aim of this study was to reveal the effect of the CF’s treatment by low-temperature plasma with runaway electrons on the deformation behavior of the PEEK-layered composites in the framework of the experimental and theoretical studies. The effect of the interlayer adhesion on the mechanical response of the composites was assessed through the tensile tests (1) and the three-point bending (2). In addition, computer simulations of the three-point bending were carried out with the use of the FEM with varying conditions at the interface of the PEEK–CF layers.

## 2. Materials and Methods

### 2.1. Experimental Study

The 250 μm thick PEEK film (Aptiv 2000, Victrex, UK) was used as a thermoplastic binder. Unidirectional CF tapes 12K-300-230 (UMATEX, Moscow, Russia) with a surface density of 230 g/m^2^ and a tensile strength of above 4.9 GPa were used for reinforcement. The fibers were covered with a commercial sizing agent aimed at thermosetting binders. The ratio of the components for the laminates’ fabrication (40 vol. %—CF, 60 vol. %—PEEK–binder) was identified through the literature analysis and the results of the previous studies by the authors [45,46,47].

**Figure 1:**
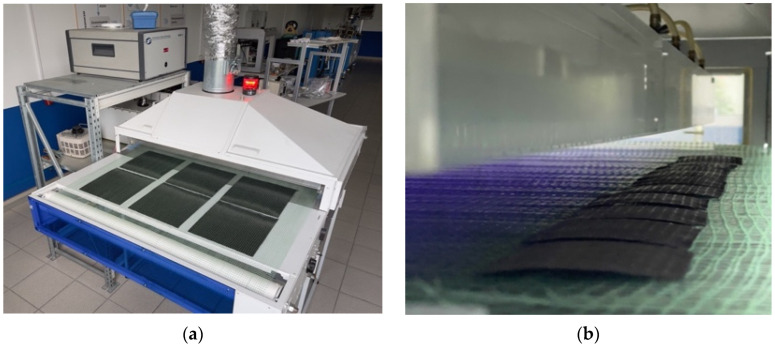
General view of the installation for cold plasma treatment of a CF tape (**a**), plasma treatment process appearance (**b**).

The installation for material treatment with low-temperature plasma of atmospheric discharge with the runaway electrons (atmospheric Discharge with Runaway Electrons—DRE) was employed for modification the CFs’ surface [48,49,50]. Figure 1 presents the general view of the installation and the CF treatment process appearance. In order to treat fibrous materials, they were placed layer-by-layer on the installation’s conveyor belt that was moving under the complex “grid” of electrodes. In this study, the CF tapes were subjected to treatment with their single-layer placement on the conveyor. The distance from the CF to the upper “complex” electrode was 50 mm. The complex high-voltage electrode consisted of three rows of steel needles shifted relative to each other at a distance of 1/3 from the one between the adjacent needles (in the perpendicular direction to the movement of the treated materials through the plasma tunnel). Each needle represents a point of stable spatial fixation of each of the DRE channels. By doing so, it provides object treatment within an individual channel. The distance between the needles of the high-voltage complex electrode was 6–12 mm. This design of the “complex” electrode and the emerging plasma discharge allows efficient and uniform treatment of fibrous materials over the entire surface by the runaway electrons. The movement of the conveyor belt was switched to the left/right so that the treated material was constantly located in the interelectrode zone.

Low-temperature DRE–plasma treatment was carried out in the mode of continuous pulse generation with the following characteristics: pulse amplitude of output voltage of 56 kV, pulse front duration of 10 ns, and pulse duration at a half-height of 40 ns. The choice of this mode was underpinned with the experience gained through a variety of treatment studies of fibrous materials [51]. The only variable parameter was the time for the CF exposure in the plasma channel, which was 5, 10, and 15 min when subjected to one side of the tape. The CF tapes were treated sequentially on each side. The topography of the plasma treated CFs was carried out using an optical interferential profilometer New View 6200 (“Zygo Corporation”, Middlefield, CT, USA).

**Figure 2:**
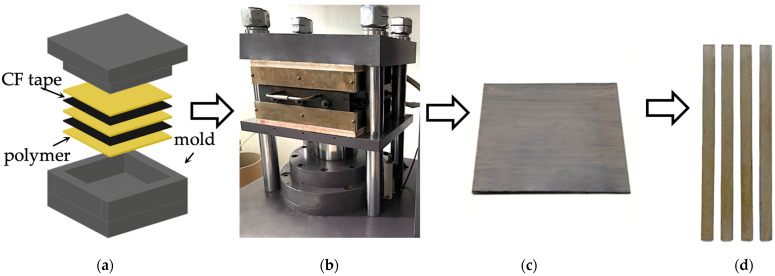
Composite work piece fabrication: laying of the layers (**a**); hot pressing (**b**); blanks for cutting the samples for mechanical tests (**c**); end samples (**d**).

The rectangular shape scissor-sliced fragments of the PEEK film, as well as of the CF tape, were alternately laid in a mold (Figure 2a). The total number of layers was 25 (13 layers of the PEEK film and 12 layers of the CF tapes; the outer layers were from the PEEK film). Sintering was performed in a GT-7014 thermohydraulic press (GOTECH Testing Machines Inc., Taiwan) (Figure 2b). The sintering temperature was 400 °C, the pressure was 6.5 MPa, and the hold time under pressure was 30 min. The workpieces were cooled down at a pressure of 4 MPa and at a cooling speed of 2 °C/min (Figure 2b). The workpieces turned to have the shape of the rectangular tiles (Figure 2c), the size of which was 270 × 250 × 4 mm. The samples for mechanical test of the specified dimension were cut by a computer numerical controlled (CNC) milling machine (Figure 2d).

The interlayer shear tests (short beam bending) were carried out according to ASTM D2344M-16. An Instron 5582 Electromechanical Testing Machine (Instron Inc, Norwood, MA, USA) was used. The cross-head speed was 1 mm/min. The sample size was 24 × 8 × 4 mm. The flexural strength at the composites was determined under three-point bending according to ASTM D7264M-15. The sample size was 153 × 13 × 4 mm. The cross-head speed was 1 mm/min.

The tensile tests were performed according to ASTM D3039M-14. A universal servo-hydraulic testing machine UTM 100kN (Biss, Bangalore, India) was employed. The cross-head speed was 2 mm/min. The test coupons had a rectangular shape with the size of 250 × 15 × 4 mm. When fixing the samples, the initial distance between the grips was equal to 150 mm.

The number of the samples of each type was at least four.

Visual examination of the fractured samples after the three-point bending tests was performed by Neophot 2 optical microscope (Carl Zeiss, Jenna, Germany). The structure over the cross-section and the cracking patterns were examined. The analysis of the fiber surface and the structure of the polymer laminates were performed by a scanning electron microscope Quanta 200 3D (FEI Company, Hillsboro, OR, USA) equipped with a focused beam system at accelerating voltage of 20 kV. The copper films that were about 10 nm thick were deposited on the fracture surfaces with the help of a “JEOL JEE-420” vacuum evaporator (JEOL USA, Inc., Peabody, MA, USA). Infrared spectroscopy of the as-received and modified fibers was performed on Tensor 27 infrared Fourier spectrometer (Bruker Scientific LLC, Billerica, MA, USA), resolution 4 cm^−1^, ZnSe ATR set-top box, 128 accumulations.

### 2.2. Simulation of Three-Point Bending of Laminates

**Figure 3:**
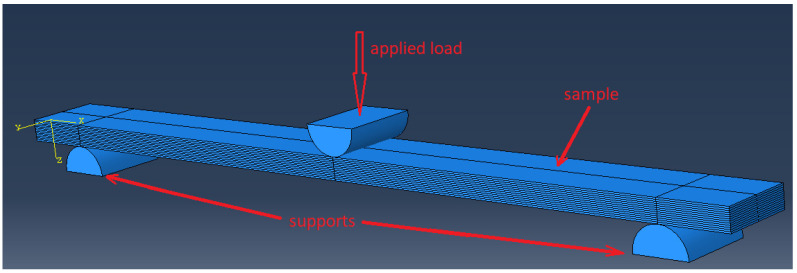
The calculation scheme for three-point bending simulation.

The theoretical study of the effect of the interfacial adhesion on the mechanical properties of the layered polymer PEEK–CF composites under the three-point bending was carried out by the FEM. The sample type and loading schemes used in the numerical simulation corresponded to the experimental ones (Figure 3). The sample length was 153 mm. The width was 13 mm and it consisted of 13 layers, and the thickness of each was 0.3 mm. The PEEK layers alternated with the CF layers along Oz direction. The centers of the supports, on which the samples were located, made the span of 128 mm. The radius of each support was 5 mm.

The contact problem of the elasticity theory was solved in three-dimensional space to determine the parameters of the deformation behavior. The FEM analysis was carried out with the use of Abaqus/CAE 2019 software package (Dassault Systems, France).

The following boundary conditions were taken.

- Lower supports were rigidly fixed.
- The load was applied through the loading roller (a cylinder) toward Oz direction, the roller’s displacements along other axes were prohibited.

The volumetric tetrahedral elements C3D10 with a quadratic approximation of displacements were used while constructing the finite element model. The sizes of the elements were selected based on the mesh convergence of the results obtained. For the sample and the supports contact cites (as well as under the loading roller), the contact conditions of the “Hard contact” were specified; these conditions allowed slipping but excluded penetration into the material. Between the layers, the contact conditions “Cohesive behavior” and “Damage” were set. These conditions did not allow mutual penetration of the layers; however, the delamination at reaching the specified level of the adhesion was allowed. Such conditions enabled us to set both the initial stiffness (K) at the interlayer boundary and the interlayer shear stress level (d).

The CF layers were modeled without explicit consideration of enforcement, but their properties (i.e., tensile moduli, flexural moduli, tensile strength) were assumed to be orthotropic. The properties were calculated under tension and shear by simulating a composite with explicit consideration of enforcement. In doing so, an assumption was made that the PEEK impregnated CF tape layer contains 80% CF and 20% binder (80 vol. % CF + 20 vol. % PEEK) [47].

Some characteristics of fiber-reinforced composites can also be calculated analytically, e.g., [52]. In this case, the longitudinal elastic modulus at the CF–polymer ratio of 80/20 vol. % was 196 GPa, while the transverse modulus of elasticity was 10 GPa. Along the Oz direction, the modulus of elasticity was assumed to be the same as along the Ox direction.

Reaching the tensile strength of the fibers was taken as their failure criterion. The tensile strength of the composite enforced with the carbon fibers under their perfect adhesion to the PEEK matrix (according to the calculations [47]) was taken equal to 4000 MPa along the direction of reinforcement, while it was 55 MPa across it.

The nonlinear pattern of the stress–strain diagram of the PEEK was taken into account; the tensile strength for normal stresses was taken equal to 108 MPa, while it was equal to 36 MPa for the shear stresses.

## 3. Experimental Results

### 3.1. Electron Microscopy and Optical Profilometry Analysis of the CFs’ Surface

**Figure 4:**
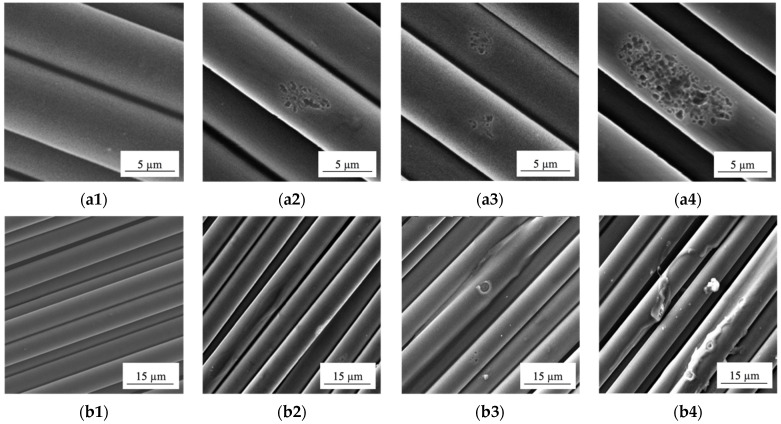
SEM micrographs of carbon fibers before (**a1**,**b1**) and after DRE–plasma treatment during: t = 5 min (**a2**,**b2**); t = 10 min (**a3**,**b3**); t = 15 min (**a4**,**b4**).

The SEM micrographs of the non-treated and the plasma-modified CFs are shown in Figure 4. The non-treated CFs, having a commercial sizing agent on the surface (Figure 4a1,b1), looked smooth, without obvious roughness. DRE–plasma treatment of CFs facilitated the formation of microroughness. This should increase the level of the adhesion (its mechanical component) at the polymer–fiber interface. With increasing the plasma treatment time (due to episodic electrical breakdowns caused the insulating properties of the sizing agent), the surface of the CFs experienced a local erosion over some of the fibers (Figure 4a2–a4). This should give rise to decreasing their strength (to be discussed below).

With increasing the time of DRE–plasma treatment, fragmentary desizing of the agent’s layer from their surface was observed (in addition to a local change in the CF roughness, Figure 4b2–b4). However, the local desizing/damage of the agent’s layer exerted a positive effect on the adhesion strength of the composites under study, since a commercial sizing agent is initially designed for thermosetting binders (epoxy resin, polyurethane, etc.). The latter did not contribute to the formation of chemical bonds between the CFs and the thermoplastic binder [53,54]. It is known that at sintering of PEEK–CF composites without desizing a finishing agent, the degradation of the latter occurs. As a result, a transition layer with reduced strength is formed at their interface.

The literature discusses the development of effective methods for preliminary desizing of a finishing agent at manufacturing of the PEEK/CF composites [9]. Factually, the above-proposed solutions on preliminary fibers annealing (oxidation) [55] could not be used in the case of the CFs tapes. When holding in the furnace at T = 400 °C, the weft thread was burned out and the integrity of the CF tape was violated (decomposed into separate fibers) when being laid in the mold. Chemical washing methods are time-consuming and environmentally unsustainable.

**Figure 5:**
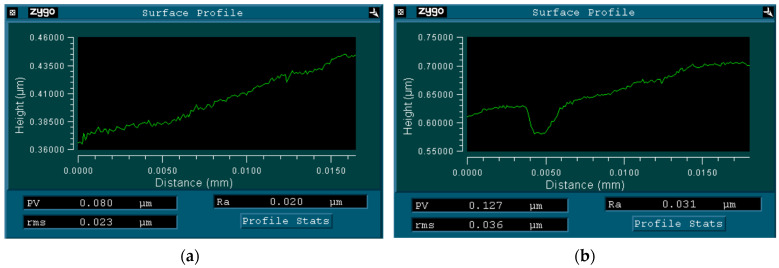
Surface profiles of CFs: untreated (**a**); treated by DRE–plasma during 15 min (**b**).

The optical interferential profilometer characteristics of the untreated and treated CFs is shown in Figure 5.

The average roughness of the untreated CFs was equal to R_a_ = 20 nm; evidently, a smooth surface was characteristic for them (Figure 5a). DRE–plasma treatment during 15 min gave rise to increasing the surface roughness up to R_a_ = 31 nm (+50%). The erosion marks were seen there, the depth of which reached R_max_ = 50 nm (Figure 5b).

### 3.2. IR Analysis of Carbon Fibers

**Figure 6:**
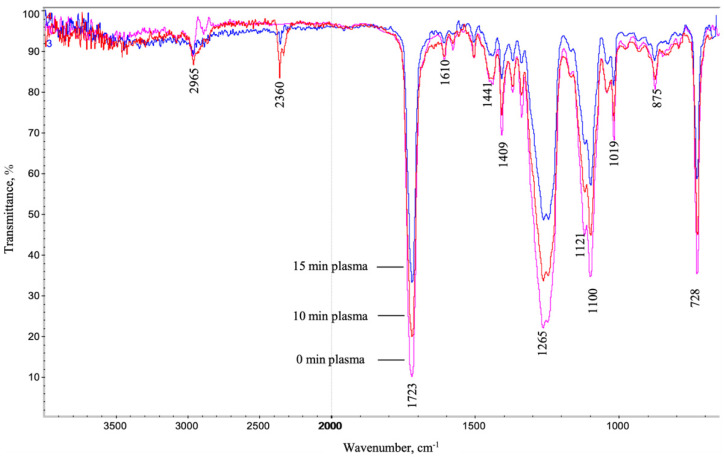
IR spectra of non-treated and DRE–plasma-modified CFs.

Infrared spectra of the CFs tapes in the non-treated state, as well as those subjected to plasma treatment for 10 and 15 min, are presented in Figure 6. Due to the high noise level, it was quite difficult to identify the individual peaks characterizing the presence of different functional groups in spectra. This was most likely due to the small thickness, and, accordingly, the small content of the sizing layer on the CFs’ surface. However, for the distinct peaks (C-H, rocking vibrations, 728 cm^−1^; C-O, stretching vibrations, 1019–1121 cm^−1^; C-O, bending vibrations, 1265 cm^−1^; C=O, stretching vibrations, at 1723 cm^−1^; C-H, stretching vibrations, at 2965 cm^−1^), there was a pronounced trend on decreasing their intensity with enlarging the DRE–plasma exposure time. In addition, this was consistent with the general principles of cold plasma fiber cleaning [18,19,20]. This was also indicated regarding the absence of new bonds formation. In addition, the decrease in the intensity of the peaks (shown with an arrow in Figure 6) could indicate precisely in favor of the partial desizing of the factory deposited commercial sizing agent with increasing the plasma treatment time.

### 3.3. Interlayer Shear Strength (Short Beam Bending)

**Figure 7:**
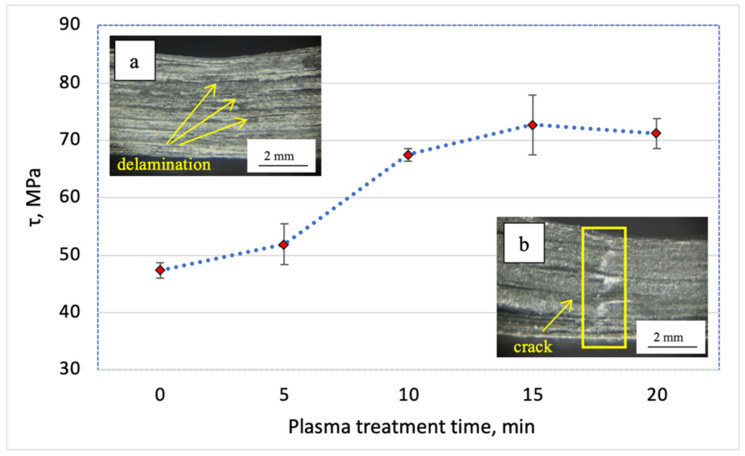
Dependence of interlayer shear strength vs. the DRE–plasma treatment time.

A short beam bending is a wide approved type for mechanical tests that more completely characterizes the level of the interlayer adhesion of laminates. At that, the value of the tensile strength under interlayer shear tests was estimated. The test results are summarized in Table 1 and Figure 7.

As shown, DRE–plasma treatment of the fibers for 5 min gave rise to the increase in the shear strength of ~10%. However, the most pronounced effect (more than 40%) was observed when the treatment time exceeded ≥10 min. The maximum increase was achieved when the time of the plasma modification was t = 15 min, which allowed the fibers to attain the level of the shear strength of ~73 MPa (i.e., the increase was +54% as compared to the composite containing the unmodified CF). Further increasing the time of DRE–plasma treatment up to the t = 20 min was not accompanied by the increase of τ. In addition, it is economically unreasonable. Thus, only the CF–PEEK laminates with the plasma treatment time of t = 15 min were further utilized. Note that the fracture pattern of the samples has changed from typical for the laminates with a low interlayer adhesion, i.e., delamination (Figure 7a) towards the fracture with the main crack propagation (Figure 7b). The latter was an indicator of increasing the interlayer adhesion as a result of DRE–plasma treatment of the CFs.

### 3.4. Uniaxial Tension Tests

**Figure 8:**
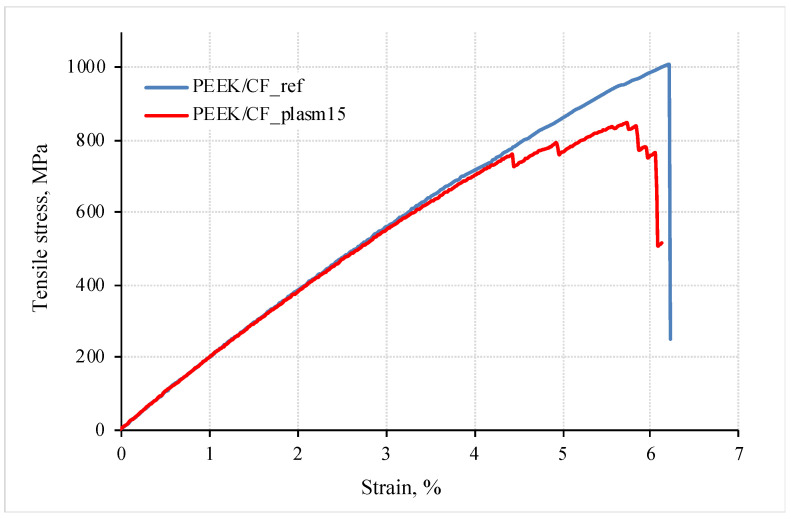
Tensile loading diagrams of PEEK/CF laminates with non-treated and DRE–plasma treated carbon fibers.

In addition to the interlayer shear tests, data of the uniaxial tensile tests (in the direction of reinforcement) are an important indication characterizing the strength of the laminates. Figure 8 illustrates the loading diagrams. The quantitative values of the mechanical properties of the studied composites are presented in Table 2.

These results testify that the proposed mode of DRE–plasma treatment gave rise to the decrease in the tensile strength (under stretching) by 18%. The values of the elongation at break were almost identical. No noticeable difference in the values of the modulus of elasticity for the samples of both types was found (up to ~19 GPa). In this case, the failure of the laminate with surface-modified CFs progressed stepwise. Similar to the results of the previous section, this may be due to the arresting of the main crack at every interlayer boundary. In the laminates with non-treated CFs, the failure pattern was brittle (Figure 8).

**Figure 9:**
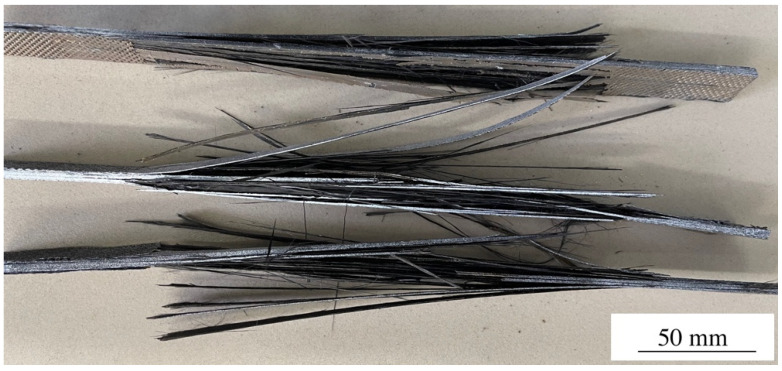
Optical photographs of fractured laminates (one in the top—with plasma treated carbon fibers; two at the bottom—non-treated CFs).

However, during the breakage, the laminates underwent a complete destruction. As a result, no visual difference between the fractured samples could be revealed (Figure 9).

As far as the test results have shown, the CF modification was accompanied by the development of local erosion on the fiber surface (Figure 4a2–a4). Each damage was a stress riser. With increasing the external load, it could give rise to a local fracture of the reinforcing carbon fibers.

### 3.5. Three-Point Bending Test

**Figure 10:**
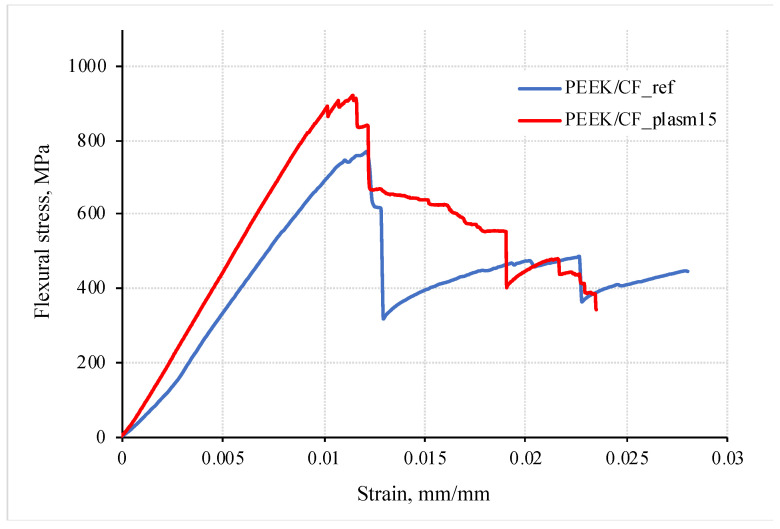
The flexural stress–strain diagram of PEEK/CF laminates without and after DRE–plasma treatment of CFs during 15 min.

Figure 10 shows the engineering stress–strain diagrams (dependence of the bending stress vs. the bending strain) of the studied composites for the non-treated and DRE plasma-modified CFs (at t = 15 min). The mechanical properties of the samples are presented in Table 3. It was found that DRE–plasma treatment of the CFs resulted in the increase in the flexural strength and flexural modulus by 16% (up to 893 MPa) and 10% (up to 93 GPa), respectively. Moreover, at the onset of main crack initiation, there was a ~58% drop in the level of a deformation stress (from 770 MPa to 320 MPa), while in the case of the modified CFs, it did not exceed 27% (from ~890 MPa to 650 MPa).

**Figure 11:**
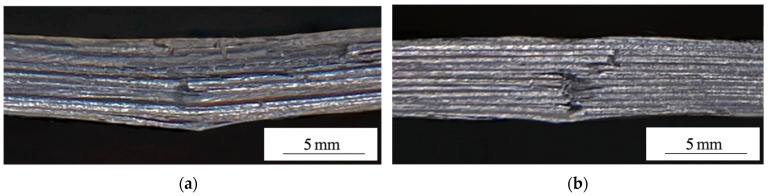
Optical images of PEEK/CF laminates fractured under three-point bending test; non-treated CF (**a**); DRE–plasma modification during t = 15 min (**b**).

This effect was related to the difference in the pattern of the macrodefect’s (main crack) propagation (see Figure 11, where the micrographs of the fractured samples after the end of the three-point bending test are presented). Figure 11a shows that in the laminate containing the non-treated CFs, the numerous discontinuities along the interlayer boundaries (a polymer layer/CF tape) were developed in the area under the loading roller that was caused by the shear stresses. DRE–plasma treatment resulted in increasing the level of the interlayer adhesion. This firstly prevented the development of an interlayer delamination; secondly, it propagated towards the normal to the surface (the main crack emerged from the opposite to the point where the load was applied). However, the macroscopic fracture was suppressed at each interlayer boundary located along the normal to its propagation path (Figure 11b). This led to the markedly smaller decrease in the level of a deformation stress during its development (see Figure 10).

### 3.6. Structural Studies

SEM–micrographs of the bending surfaces of the PEEK/CF composites are shown in Figure 11. The key issue for their analysis was to identify the interaction patterns of the binder with the reinforcing fibers, as well as the effect of DRE–plasma surface treatment.

**Figure 12:**
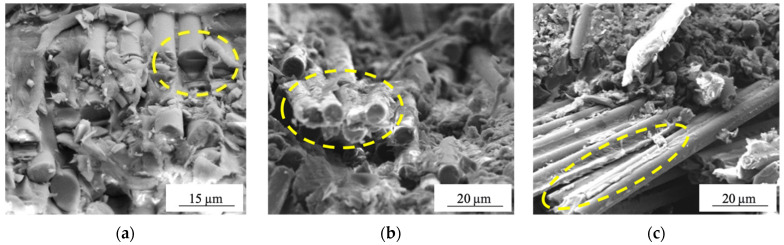
SEM micrographs of internal structure of composites: (**a**) PEEK/CF (untreated); (**b**) PEEK/CF (t_plasm_ = 10 min.); (**c**) PEEK/CF (t_plasm_ = 15 min).

In the non-modified CFs enforced laminate, the surface of the fibers did not contain any binder fragments (shown by the oval). This indicated insufficient wetting and, accordingly, a low adhesion level (Figure 12a). Under rupture, the fracture occurred along the “fiber/polymer” interlayer/interphase boundaries. In the case of DRE–plasma modification, the CF surface was better wetted with the PEEK binder (Figure 12b, shown by the oval). This allowed it to preserve its fragments on the surface of the fibers (Figure 12c, shown by the oval).

## 4. Numerical Simulation

In order to conduct detailed parametric studies on the effect of the interlayer adhesion over the mechanical properties of the composites, the theoretical studies were carried out based on the FE modeling.

The strain and stress of the composites under bending were determined by the Formulas (1) and (2):

(1) $$\varepsilon =\frac{6hy}{L^{2}}$$

where *y* is the deflection of the neutral axis, *h* is the height of the sample, and *L* is the span (distance between the supports);

(2) $$\sigma =\frac{3LF}{2bh^{2}}$$

where *F* is the applied force and *b* is the sample width.

Within this statement, the strength of the composites was determined by the delamination stress and the initial interlayer stiffness. For this reason, the effect of these parameters on the strength of the composites was assessed below.

DRE–plasma treatment of the CFs changed their strength and the adhesion to the polymer. In the simulation, the influence of these factors was assessed by varying: (i) the initial interlayer stiffness at the contact of the layers (*K*), (ii) the effective modulus of elasticity of the layer with CFs (*E*), and (iii) the delamination stress (*d*).

**Figure 13:**
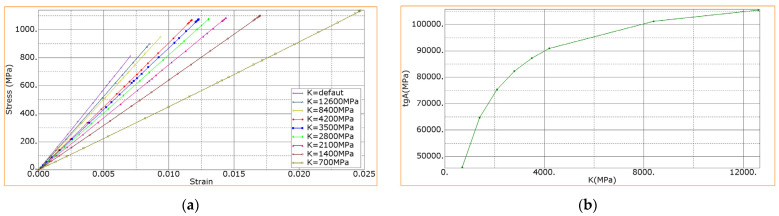
Dependencies: (**a**) the bending stress vs. the bending strain of the composites at various levels of the interlayer stiffness; (**b**) the dependence of laminate’s flexural moduli vs. the interlayer stiffness.

The increase of the inclination angle of the stress–strain diagram at the bending of the laminates with the treated fibers (Figure 8) can be related to the increase in the interlayer adhesion. In the simulation, this, among other things, was reflected by the parameter of the initial interlayer stiffness *K*. To assess the level of the interlayer stiffness below, its value was varied from 700 to 12,600 MPa with constant values of other specified parameters. The results are shown in Figure 13.

It is seen that at the initial interlayer stiffness of *K* = 2100 MPa (Figure 13a), the inclination angle of the stress–strain curve corresponded to that of the experimentally registered dependence for the laminate with the non-modified CFs (Figure 8). At the same time, at *K* = 4200 MPa, the inclination angle of the curve corresponded to that with the DRE–plasma-modified CFs. Thus, the interlayer stiffness increased by ≈2 times.

The inclination angle of the stress–strain diagram in Figure 13a characterizes the flexural modulus and is equal to tg *a* (Figure 13b). It is seen that with increasing the initial stiffness, it raised nonlinearly. Since the initial interlayer stiffness enlarged with increasing the adhesion, the results obtained testify that with increasing adhesion a higher level of the interlayer initial stiffness might be attained. The stiffness would be limited by the one corresponding to the ideal adhesion (see the curve at *K* = default, Figure 13a).

It is seen in Figure 13 that with increasing the interlayer stiffness, with other equal parameters, the strength decreased nonlinearly in the range from 1135 MPa to 811 MPa. At the maximum stiffness (*K* = default, Figure 13a), the achieved ultimate strength was reduced down to *d* = 811 MPa. Thus, the increase in the interlayer stiffness, attained by increasing the adhesion, gave rise to the decreasing the strength. The reason was as follows: when the interlayer stiffness was large, the deformability of the sample decreased, and the ultimate strength was reached faster. In this regard, it is important to choose the “golden mean”, that is, to select such an adhesion at which both the flexural modulus and strength will increase simultaneously. In Figure 13a, these requirements correspond to curve *K* = 4200 MPa; as seen, a further increase in the interlayer stiffness resulted in decreasing the strength.

In the case of DRE plasma treatment of the CF, the tensile modulus of the composite and its strength has changed (Figure 8). Moreover, these parameters could be increased by enlarging the adhesion level. In addition, they can be reduced due to damaging the CF. This, in turn, gave rise to changing the pattern of the stress–strain diagram at bending (Figure 8). It is seen in Figure 8 that the inclination angle of the stress–strain diagram practically did not change. This indicated that the elastic modulus of the composite remained practically constant.

To assess the dependence of the flexural modulus as well as composites’ strength vs. the effective tensile moduli of the CF layer, the longitudinal modulus of elasticity of the CF layer was varied in the range from 49 to 196 MPa. Since the layer possessed the orthotropic properties, the transverse moduli of elasticity under tension along y and x axes varied in proportion to the change in the longitudinal modulus of elasticity. In this case, the initial stiffness (*K*) and the limit of the delamination stresses of the layers (*d*) remained constant: *K* = 4200 MPa, *d* = 45 MPa (corresponding to the experimental results).

**Figure 14:**
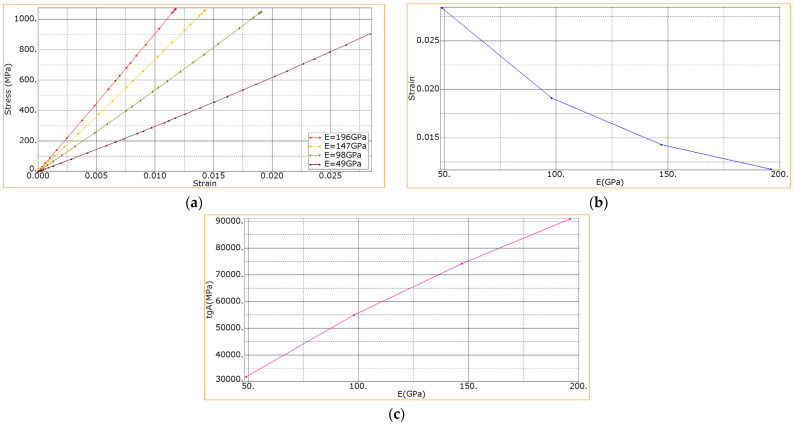
Dependencies of: (**a**) the bending stress vs. the relative bending strain at variation of the elastic modulus of the CF layer; (**b**) the ultimate strain vs. the elastic modulus of the CF layer; (**c**) the flexural modulus of the composite vs. the elastic modulus of the CF layer.

Figure 14a shows the calculated dependences of the flexural stress vs. the relative flexural strain when varying the effective elastic moduli of the CF layer. It is seen that the decrease in the tensile modulus of the CF layer also led to the decrease in the flexural modulus of the composite, while the strength of the composite also decreased (Figure 14a). Simultaneously, the failure strain of the composite (Figure 14b) decreased nonlinearly. In addition, the flexural modulus increased nonlinearly (see the inclination angle in Figure 14c). Note that when modeling, the nonlinearity of these dependencies can be induced by the nonlinearity of the stress–strain diagram under tension for the PEEK layers.

Further, the effect of the layer delamination stress (*d*) on the mechanical properties of the laminate in the range from 15 to 75 MPa was analyzed. In this case, the tensile modulus of the layers (*E*) and the initial stiffness (*K*) remained constant: *E* = 196 GPa, *K* = 4200 MPa, corresponding to the dependence that was obtained experimentally (Figure 8).

**Figure 15:**
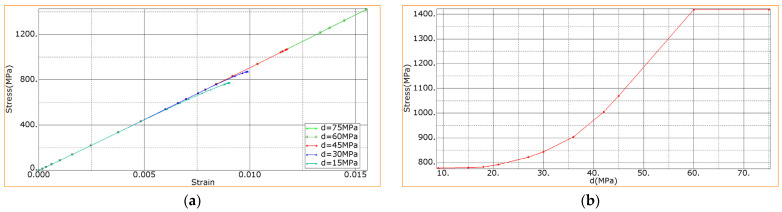
Dependencies: (**a**) the bending stress vs. the bending strain at various level of the interlayer adhesion; (**b**)—the ultimate strength vs. the level of the interlayer adhesion.

Figure 15 shows the calculated dependences of the bending stress vs. the relative bending strain of the composites with a variation of the delamination stresses of the layers. It is seen that with a higher level of the adhesion, a higher strength of the composites was attained (Figure 15a); however, exceeding the delamination stresses over 60 MPa did not lead to any increase in strength—the dependencies for *d* = 60 and *d* = 75 MPa coincided. Comparison of the calculated and experimentally-registered dependencies for the sample with the CFs subjected to DRE–plasma treatment (see the previous section of Figure 8) corresponded to the delamination stress level of approximately 40 MPa. This value can be used as an “indicative” value when assessing the achieved level of the interfacial adhesion in the experimental study. It is evident that for the composites under consideration, a higher strength up to 1400 MPa can be “theoretically” obtained (Figure 15a).

The dependencies shown in Figure 15a are linear, but each curve ends with a non-linear section. On the one hand, this was due to the initiation of fracture (due to the interlayer shear and/or cracking in the material); on the other hand, this was due to the nonlinearity of the stress–strain diagram for the PEEK. Thus, the dependence in Figure 15b was due to the type of the fracture onset. For a *d* value less than 20 MPa, the failure occurred mainly due to the interlayer shear, which was caused by the weak interlayer adhesion. Next, a crack was nucleated. When Abaqus software was used, the appearance of a crack terminated the calculation (equivalent to the reaching of the fracture criterion). With the increase of *d* from 20 to 60 MPa, the fracture began later, but this was also due to the interlayer shear (then a crack emerged as well). For cases of *d* above 60 MPa, there was no interlayer shear, and the crack started from the upper face from the CF layer; in doing so, the ultimate strength at *d* above 60 MPa did not change.

**Figure 16:**
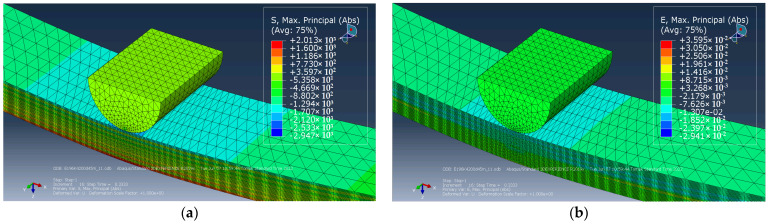
Distribution of stresses (**a**) and strains (**b**) in a CF/PEEK laminate under three-point bending.

Figure 16a shows that the highest tensile stresses were developed in the center of the sample in its lower layers. The highest compressive stresses were localized under the loading roller on the upper layers of the sample and concentrated in the CF layers (Figure 16a). The greatest strains were also revealed in the center of the sample and reduced within the polymer layers (Figure 16b). It is seen in Figure 16 that the compressive stress under the loading roller at the top of the sample was higher than the tensile ones at its bottom part. For this reason, the crack nucleated under the loading roller in the upper CF layer in the center of the sample and developed along the y and z axes (parallel to the direction of load application). Its view, dimensions and location are described in Figure 16b.

Thus, on the one hand, it was shown that the increase in the values of the considered parameters exerted an ambiguous effect on the mechanical properties of the laminates at three-point bending. On the other hand, theoretically, the strength of the laminate could be raised by increasing the interlayer adhesion.

It should be noted that the model, which was used, does not imply the depiction of the complete fracture of the composite, although this was one of the important factors determining the load-bearing capacity of the composite. In forthcoming research the authors intend to improve the model, which will take this factor into account.

## 5. Discussion

The obtained results compared with the literature ones are summarized in Table 4.

Note that the majority of research papers are mostly focused on the CF/epoxy composites. In addition, various plasma sources have been employed. The current study was aimed at DRE–plasma treatment of the CF tapes for fabricating thermoplastic laminates.

Before concluding, the authors would like to cite the relevant paper by Cho et al. [29]: “The current level of properties of conventional composites is determined by the results of a half a century optimization. However, the multiscale hybrid composites are still an open field requiring further systematic studies”.

## 6. Conclusions

The layered PEEK–CF laminates fabricated by film stacking were studied, in which the interlayer adhesion was increased by DRE plasma treatment of the CF tapes with a different time exposure.

The study of the structure of the modified fibers by scanning electron microscopy have shown that DRE–plasma treatment during the optimal time of t = 15 min has led to the formation of a more developed (rough) surface. There also was a partial desizing a commercial agent from the surface of the CF, which is confirmed by the IR–spectroscopy data. The analysis of the laminates’ fracture surfaces has shown the increase in the level of the interfacial adhesion between the CF and the PEEK, which was manifested in the preservation of the binder fragments on the surface of the treated fibers.

The analysis of the strain behavior has shown that DRE plasma modification of the surface of the CFs within 15 min has led to the increase in the shear strength of the layered composites by 54% (up to ~73 MPa); the tensile strength and flexural modulus (at three-point bending) have increased by 16% (up to 893 MPa) and by 10% (up to 93 GPa), respectively. However, there has also been an 18% reduction in the tensile strength (up to 834 MPa), which was associated with the development of a local erosion of the fiber surface during the treatment used.

On the basis of the developed finite element model, the influence of parameters such as the initial stiffness at the interlayer boundary, the tensile modulus of the CF layer, and the delamination stress of the layers onto mechanical properties of the composites under bending were analyzed. In addition, the simulation has allowed us to assess the influence of these factors on the type of composites’ failure. Comparison of the simulation results with the experimentally obtained ones has shown that the stress level achieved in the experiments corresponded to ~40 MPa. This value can be used as an indicative level of the applied interfacial adhesion. It has been shown that the increase of the delamination stress of the layers up to 60 MPa enabled the avoidance of an interlayer failure and increased the flexural strength of the laminate up to 1400 MPa.

The analysis of the results of the numerical experiments, when the interlayer stiffness was varied, has shown that the increase in the stiffness, on the one hand, gave rise to the increase in the flexural modulus; on the other hand, there may be a nonlinear decrease in strength. For this reason, the intention to maximize the level of the interlayer stiffness is not always appropriate, for example, in cases of manufacturing high-strength composites. This is also an important factor that determines the resistance of structures to the main cracks’ development (as a parameter proportional to the fracture toughness).

## Figures and Tables

**Table 1:** The interlayer shear strength (under bending of the short beam) of the PEEK-based composites reinforced with unidirectional CF tapes.

PEEK/CF Composite Type	Ultimate Shear Strength, MPa	Increase, %
without treatment	47.5 ± 1.4	-
t_plasm_ = 5 min	51.9 ± 3.6	+10
t_plasm_ = 10 min	67.5 ± 1.1	+43
t_plasm_ = 15 min	72.7 ± 5.2	+54
t_plasm_ = 20 min	71.2 ± 2.7	+51

**Table 2:** Mechanical properties of CF–PEEK laminates at static tension.

Sample	Tensile Modulus, GPa	Ultimate Tensile Strength, MPa	Ultimate Tensile Strain, mm/mm
PEEK/CF_ref_	18.5 ± 1.7	1016 ± 106	0.063 ± 0.003
PEEK/CF_plasm15_	18.8 ± 1.1	834 ± 52	0.062 ± 0.004

**Table 3:** Mechanical properties of CF–PEEK laminates under three-point bending.

Sample	Flexural Modulus, GPa	Ultimate Flexural Strength, MPa	Ultimate Flexural Strain, mm/mm
PEEK/CF_ref_	82.6 ± 2.4	770 ± 63	0.012 ± 0.01
PEEK/CF_plasm15_	92.6 ± 2.3	893 ± 41	0.011 ± 0.02

**Table 4:** The summary on CFs’ plasma treatment effect versus some physical–mechanical properties of both fibers and composites.

Composite	Treatment Parameters	Variation of Mechanical Properties after Plasma Treatment	Reference
CF/epoxy	Vacuum 0.1–0.001 mm Hg, RF–plasma, 0–300 W, 20 MHz	ILSS: +67% Tensile strength of single CF: −1.6%	Mujin1989 [19]
CF/PC	Low-pressure (100 Pa), oxygen microwave plasma, 2450 MHz, 75 W, 3 min	Tensile strength of single CF: +280% (ultra-high modulus CF), +12% (high strength CF)	Montes-Morán 2001 [28]
CF/PPESK	Air (60–80 cm^3^/min) Inductively coupled plasma (ICP), 250 W, 5/10/15/20 min	ILSS: +14% (15 min)	Lu 2007 [35]
CF/PES, CF/PEI, CF/PEEK	N_2_ or N_2_ + O_2_ (33 m^3^/h flow rate) microwave plasma 2450 MHz, 1200 W	ILSS up to: +55% (CF/PES), +32% (CF/PEI), +7.5% (CF/PEEK)	Tiwary 2010 [31]
CF/epoxy	Vacuum 15 Pa, oxygen (flow rate 6–8 sccm) RF–plasma, 13.56 MHz, 300 W, 3/5/7 min	ILSS: +23% (7 min) Flexural strength: +3% (7 min)	Ma 2011 [36]
CF/PI	He (20 L/min) + O_2_ (0.4 L/min) RF–plasma, 13.56 MHz, 100 W, 2.5 mm/s, 60 °C, 16/32/64 s	IFSS: +20% (32 s) Tensile strength of single CF: −2% (64 s)	Xie 2011 [26]
CF/PP	Air DBD plasma, 2/5/10 min	ILSS: +67% (5 min)	Santos 2013 [37]
CF/epoxy	Oxygen (flow rate of 100 sccm) RF–plasma 125 W, 1 min	ILSS: +28% Tensile strength of single CF: +/−0	Baghery Borooj 2016 [33]
CF/geopolymer	Ar/Ar + N_2_/Ar + O_2_/Ar + water vapor (50 L/min Ar, 0.5 L/min admixture), RF–plasma,13.56 MHz, 150 W, 2 m/min	ILSS: +24% (Ar)	Diblíková 2019 [38]
CF/epoxy	Low-pressure (<1 × 10^4^ Pa) RF–plasma, 13.56 MHz, 100 W, (airflow < 5 cm^3^/min STP), 180/300 s. Air DBD–plasma, 300 W, 10–20 kHz, 20 kV, 10/60 s	IFSS: +360% (RF), +620% (DBD) Tensile strength of single CF: preserved (RF, 300 s), −10 ca.% (DBD, 60 s)	Karoly 2021 [39]
CF/epoxy	Low-pressure (40 Pa), oxygen plasma, 13.56 MHz, 100/200 W, 5 min	Tensile strength of single CF: +23% Tribology +33%Surface hardness +11%	Khan 2021 [40]
CF/epoxy	Air RF–plasma, 13.56 MHz, 5–100 W, 5 min	IFSS: +394% (20 W)	Radjef 2021 [42]
CF/epoxy	Air (0.7 MPa, 2.5 L/min) DBD–plasma, 600 W, 7.5/15/30 s.	ILSS: +23% (60 s)	Xiao 2022 [41]
CF/PEEK	Air DRE–plasma, 56 kV, pulse front duration of 10 ns, pulse duration at half-height of 40 ns, 5/10/15/20 min	ILSS: +54% (15 min) Tensile strength: −17% (15 min) Flexural strength: +16% (15 min)	[Current study]

## Data Availability

The data presented in this study are available on request from the corresponding author.
